# Comparison of Clinical Outcomes of Two-Level PELD and Foraminoplasty PELD for Highly Migrated Disc Herniations: A Comparative Study

**DOI:** 10.1155/2019/9681424

**Published:** 2019-10-13

**Authors:** Xinbo Wu, Guoxin Fan, Shisheng He, Xin Gu, Yunfeng Yang

**Affiliations:** ^1^Department of Orthopedics, Shanghai Tongji Hospital, Tongji University School of Medicine, Shanghai 200065, China; ^2^Department of Orthopedics, Shanghai Tenth People's Hospital, Tongji University School of Medicine, Shanghai 200072, China; ^3^Department of Orthopedics, Changzheng Hospital Affiliated to the Second Military Medical University, Shanghai 200003, China

## Abstract

**Objective:**

The aim of this study is to compare the clinical outcomes of two-level percutaneous endoscopic lumbar discectomy (PELD) and foraminoplasty PELD in treating highly migrated lumbar disc herniations.

**Methods:**

Patients with highly migrated lumbar disc herniations were enrolled from May 2014 to June 2016. Low back pain and leg pain were evaluated by the Visual Analog Scale (VAS), and functional outcomes were assessed with the Oswestry Disability Index (ODI). The satisfaction rate of clinical outcomes was assessed according to the modified MacNab criteria. In addition, the intraoperative duration and postoperative complications were also recorded.

**Results:**

Forty patients, 14 cases in two-level PELD group and 26 cases in foraminoplasty PELD group, were included. The VAS scores of low back pain (*P*=0.67) and leg pain (*P*=0.86), as well as the ODI scores (*P*=0.87), were comparative between two-level PELD and foraminoplasty PELD groups. The satisfaction rate of clinical outcomes based on the modified MacNab criteria in the two-level PELD group was equivalent to that in foraminoplasty PELD group (92.9% versus 92.3%, *P*=0.92). In addition, the intraoperative duration of two-level PELD group was longer than that of foraminoplasty PELD group (80.2 ± 6.6 min versus 64.1 ± 7.3 min, *P* < 0.01). The postoperative complications in the two-level PELD group (postoperative dysesthesia: *N* = 1) were relatively fewer as compared to those in the foraminoplasty PELD group (postoperative dysesthesia: *N* = 1; recurrence: *N* = 1; nucleus pulposus residues: *N* = 1).

**Conclusions:**

Both two-level PELD and foraminoplasty PELD are safe and effective surgical procedures for the patients with highly migrated lumbar disc herniations. Moreover, the two-level PELD technique has merits in reducing the incidence of postoperative nucleus pulposus residue.

## 1. Introduction

Lumbar disc herniations (LDH) are one of the most common causes of lower back pain and sciatica. 70%–85% of people suffered at least one episode of lower back pain with or without leg pain during their lives [1, 2, 3]. Generally, surgical treatment is necessary when conservative treatment fails. The commonly accepted surgical methods include conventional open surgery and minimally invasive surgery.

Kambin introduced the concept of indirect decompression of the spinal canal via the posterolateral approach in 1973 [4]. Hijikata firstly described the percutaneous discectomy in 1975 [5]. Since then, the techniques of percutaneous endoscopic lumbar discectomy (PELD) had been rapidly developed. It has been proved that the technique of PELD can effectively remove the herniated nucleus pulposus, can release nerve roots through the intervertebral foramen, and can achieve comparative clinical outcomes as the conventional open surgery [3, 6]. Moreover, there are many advantages of using the PELD technique, such as shorter operating time, less blood loss and postoperative pain, and faster postoperative rehabilitation and preservation of normal paraspinal structures, which contribute to a lower incidence of iatrogenic instability [7, 8, 9, 10]. Therefore, PELD has been regarded as an alternative to conventional open surgery for lumbar disc herniations [6, 11, 12].

Indications of PELD have been limited to nonmigrated or low-migrated lumbar disc herniations due to anatomical barriers that challenge its application for highly migrated disc herniations [7, 13, 14]. However, with the development of techniques and instruments, such as wide application of reamer kits, high-speed endoscopic drills, and flexible curved forceps in clinical practice, the indications of PELD have been extended to highly migrated disc herniations [15, 16, 17, 18, 19, 20]. Furthermore, in order to improve clinical outcomes and reduce the incidence of complications, various modified PELD techniques have been introduced, e.g., the foraminoplasty and interlaminar approaches [13, 21, 22]. Specifically, we have developed a new PELD technique for the treatment of highly migrated disc herniations via a novel two-level approach [19].

However, the differences of clinical outcomes between foraminoplasty PELD and two-level PELD remain largely unknown. This gap of knowledge makes it difficult to ascertain which surgical procedure is more effective for highly migrated lumbar disc herniations. We therefore set up a comparative study using a cohort of patients who had highly migrated lumbar disc herniations undergoing foraminoplasty PELD or two-level PELD. The objective of this study was to systematically compare the clinical outcomes and postoperative complications of these two techniques.

## 2. Materials and Methods

### 2.1. General Information

This study was approved by the Institutional Ethical Committee of local hospital, and all patients signed the informed consent. We conducted a retrospective analysis of 40 patients who had lower back and leg pain due to highly migrated lumbar disc herniations and underwent two-level PELD or foraminoplasty PELD by an experienced surgeon (X. G.) between May 2014 and June 2016. Basic demographic and clinical characteristics of the participants were collected, including age, sex, conservation time, follow-up time, location of lesions, and intraoperative duration. Routine computed tomography (CT) and magnetic resonance imaging (MRI) scan were conducted to confirm the level of lesions before surgery. All participants were able to complete the follow-up assessments via phone or outpatient recheck.

### 2.2. Inclusion and Exclusion Criteria

Participants were included if they met the following eligibility criteria: (1) complaining of low back and lower limb pain or numbness due to highly migrated lumbar disc herniations, (2) positive straight leg raising test (<60°) and positive augmentation test, (3) unsuccessful conservative treatment for more than 3 months, and (4) high-caudal or high-cranial soft migrated disc herniations confirmed by MRI and CT. The exclusion criteria were (1) evident disc calcification confirmed by CT, (2) recurrent disc herniations after open discectomy or PELD, (3) L1/2 or L2/3 highly migrated disc herniations, (4) high-cranially migrated herniations at L3/4 level, (5) infection, or (6) multilevel disc herniations. Specifically, high-caudally migrated herniations at the L5/S1 level were a contraindication only for the two-level PELD technique.

### 2.3. Classification of Migrated Disc Herniations

As Lee reported [23], disc migration was classified into the following four zones according to the direction and distance from the disc space: (1) High-cranial migration: from the inferior margin of the upper pedicle to 3 mm below the inferior margin of the upper pedicle. (2) Near-cranial migration: from 3 mm below the inferior margin of the upper pedicle to the inferior margin of the upper vertebral body. (3) Near-caudal migration: from the superior margin of the lower vertebral body to the center of the lower pedicle. (4) High-caudal migration: from the center to the inferior margin of the lower pedicle.

### 2.4. Clinical Assessments

Clinical data were collected before operation and at postoperative three months and twelve months and at the final follow-up. The back pain and leg pain were measured by using Visual Analog Scale (VAS), with the score ranged from 0 point (no pain) to 10 points (worst pain). Functional outcome was assessed with the Oswestry Disability Index (ODI). Besides, the intraoperative duration and postoperative complications were also recorded and analyzed. The satisfaction rate of clinical outcomes was assessed according to the modified MacNab criteria [24]. Specifically, “excellent outcome” was defined as no pain and no limitation of normal life; “good outcome” as occasional pain or paresthesia, but no need for medication and no limitation of normal life; “fair outcome” as pain relief but in need of medication and having some limitation of normal life; “poor outcome” as no improvement or even worsen pain, and need of additional operation due to incomplete decompression. In the current study, “excellent outcome” and “good outcomes” were considered to be “clinically satisfactory.” In addition, MRI examinations were conducted for all patients after the surgery to ensure the herniated nucleus pulposus had been removed successfully.

### 2.5. Surgical Techniques

#### 2.5.1. Foraminoplasty PELD Technique

PELD was performed under local anesthesia with the patient in a prone position on a radiolucent table using C-arm fluoroscopy. Patients were routinely informed about all PELD procedures. Depending on the patient's weight and surgical level, the entry point was selected 12–16 cm from the midline at the L5/S1 level, 11–14 cm at the L4/5 level, and 10–12 cm at the L3/4 level. After infiltration anesthesia of the puncture pathway with lidocaine (1%), an 18-gauge needle was inserted under the fluoroscopic guidance. The target point of the needle was the medial pedicle line on the anteroposterior view and the posterior vertebral line on the lateral view. After the needle was located to the target point, a guide wire was inserted through the needle and sequential dilators of increasing diameter were introduced along the guide wire to widen the soft tissue channel. Then, a series of reamers were used to remove the partial ventrolateral area of the superior articular process to enlarge the foramen. Then, a tapered cannulated obturator was inserted along the guidewire, and a working channel was directly placed near the herniations spot. After the obturator was removed, an endoscope was inserted through the working channel. The herniated disc was removed using endoscopic forceps and flexible bipolar radiofrequency probe. After the herniated disc was completely removed, the endoscope and working channel were withdrawn and the skin was sutured.

#### 2.5.2. Two-Level PELD Technique

**Figure 1:**
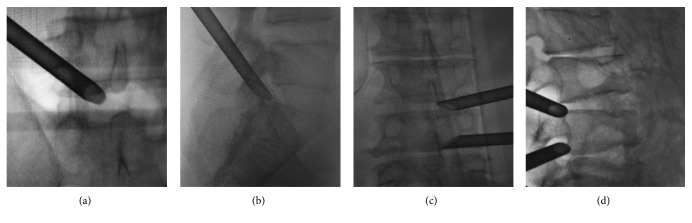
Surgical procedures of two-level PELD and foraminoplasty PELD. (a, b) Working channels placed into the intervertebral foramen at the L5/S1 level. (c, d) Two-level working channels placed into the intervertebral foramen at the L3/4 and L4/5 level.

Preoperative location and anesthesia were conducted according to the above-described procedure. The PELD was performed according to the standard procedure described by Wu et al. [19]. Here, the two-level PELD technique was described for the treatment of patients with the highly upward migrated disc herniations at L4/5 as an example. After preoperative preparation and anesthesia, an 18-gauge needle was inserted to the L4/5 intervertebral foramen under the fluoroscopic guidance. Anteroposterior fluoroscopy was used to confirm the needle positioned on the edge connection pedicle. Lateral fluoroscopy was used to confirm the needle positioned above the vertebral foramen. After a guide wire was inserted, a small skin incision was made around it. Sequential dilators were placed along the guide wire to widen the soft tissue channel. The guide wire was withdrawn and a working channel was placed into the intervertebral foramen, and the endoscope was introduced. After removing the free nucleus pulposus, the L4/5 working channel was remained. Then, an 18-gauge needle was localized to L3/4 intervertebral foramen under fluoroscopy, followed by the working channel slanted downward into the foramen. Intraoperative fluoroscopy was used to confirm the working channels had been entirely placed into the spinal canal, and the free residual nucleus pulposus could be seen located below the nerve roots. Curved forceps bit the herniated nucleus pulposus tissue. Finally, we reconfirmed no further remnants via two channels, and the incision had been sutured after removed the working channels (Figure 1).

### 2.6. Statistical Analysis

The Kolmogorov–Smirnov test was used to test the normality of all variables. The between-group differences were compared using Student's *t*-test for continuous variables (mean ± standard deviation (SD)) or a chi-square test for categorical variables (*n* (%)). The differences in the longitudinal changes of VAS and ODI scores between the foraminoplasty PELD group and two-level PELD group were compared by using repeated measures analysis of variance. All analyses were performed using the SPSS version 20.0 (SPSS Inc., Chicago, IL) and Prism 6 Software (La Jolla, CA). *P* values less than 0.05 were considered statistically significant.

## 3. Results

A total of 40 patients were enrolled in our study, including 14 cases in two-level PELD group (47.3 ± 13.3 years, 35.7% being male) and 26 cases in foraminoplasty PELD group (42.4 ± 9.4 years, 53.8% being male). There were no significant differences in age and sex between these two groups. The conservative treatment time (4.4 ± 1.0 months versus 4.5 ± 1.3 months, *P*=0.65) and follow-up time (18.5 ± 3.0 months versus 17.2 ± 2.7 months, *P*=0.15) were comparative between the two-level PELD group and the foraminoplasty PELD group. However, the average intraoperative duration of the two-level PELD group was relative longer as than that of the foraminoplasty PELD group (80.2 ± 6.6 min versus 64.1 ± 7.3 min, *P* < 0.01). In addition, the locations of lesion in two-level PELD group were L3/4 (*N* = 5), L4/5(*N* = 7), and L5/S1 (*N* = 2), which were similar to that in the foraminoplasty PELD group, including L3/4 (*N* = 4), L4/5 (*N* = 14), and L5/S1 (*N* = 8). The demographic and clinical characteristics are presented in Table 1.

**Figure 2:**
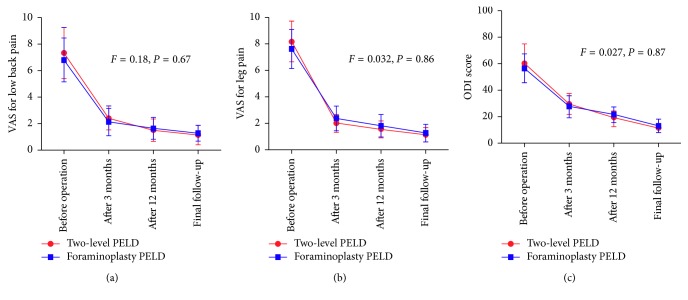
Comparisons of the improvement of VAS and ODI scores between the two-level PELD group and foraminoplasty PELD group. There were no significant differences in VAS of back and leg pain and ODI scores between the two groups. VAS: Visual Analog Scale; ODI: Oswestry Disability Index.

We firstly compared the low back and leg VAS and ODI scores between two-level PELD and foraminoplasty PELD groups before the operation and at the postoperative 3 months and 12 months and at the final follow-up, respectively (Table 2; Figure 2). Results showed that the VAS scores of low back and leg significantly decreased across the postoperative interviews after the treatment with two-level PELD (*P* < 0.01) and foraminoplasty PELD (*P* < 0.01). Similarly, the ODI scores also decreased from preoperation 60.6 ± 14.7 to 11.4 ± 3.2 at the final follow-up in the two-level PELD group (*P* < 0.01) and from preoperation 56.8 ± 11.2 to 13.2 ± 4.9 at the final follow-up in the foraminoplasty PELD group (*P* < 0.01). However, there were no significant differences in VAS (low back and leg) and ODI scores between these two groups (*P*=0.67; *P*=0.86; *P*=0.87), which demonstrated that both of these two surgical procedures could comparatively relieve the pain and improve the postoperative functional recovery.

Then, we evaluated the satisfaction rate of clinical outcomes at the final follow-up according to the modified MacNab criteria. We found that the outcomes of five patients were excellent, eight patients were good, and one patient was fair in the two-level PELD group, while the outcomes of 11 patients were excellent, 13 patients were good, one patient was fair, and one patient was poor in the foraminoplasty group (Table 2). There were no significant differences in the satisfaction rates (excellent and good) between these two surgical procedures (92.9% versus 92.3%, *P*=0.92).

**Figure 3:**
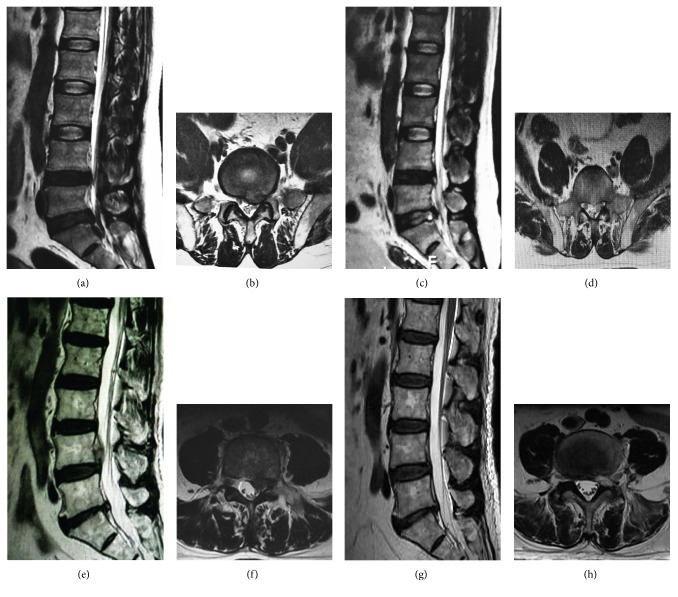
Preoperative and postoperative imaging examinations. (a, b) Preoperative MRI revealed L5/S1 disc herniations with nucleus pulposus highly downward migrated to S1 intervertebral space. (c, d) Postoperative MRI examination revealed clean removal of the nucleus pulposus. (e, f) Preoperative MRI revealed L4/5 disc herniations with nucleus pulposus highly upward migrated to L4 vertebral posterior. (g, h) Postoperative MRI examination revealed clean removal of the nucleus pulposus.

Finally, we analyzed the postoperative complications in these patients with highly migrated lumbar disc herniations after the treatment of two-level PELD and foraminoplasty PELD. The current findings suggested that the migrated disc was successfully removed and confirmed by MRI examinations after the operation (Figure 3). Generally, there were no statistical differences in the postoperative complications between these two surgical groups due to the relatively low incidence (Table 2). Specifically, both groups had one patient occurred with postoperative dysesthesia (POD), respectively; but the POD symptoms were transient and disappeared during the period of follow-up interviews. Additionally, there was one patient in the foraminoplasty PELD group recurred after the operation and received the fusion surgery and another patient had complaint of pain in legs due to residual disc which had been demonstrated by the MRI scanning and received the surgery of open discectomy. We did not find any cases in these two groups complicated with cerebrospinal fluid leak or infection during the follow-up period.

## 4. Discussion

PELD has been widely used for contained or low-migrated lumbar disc herniations, and the clinical efficacy of PELD in the transforaminal approach has been confirmed by many previous studies [3, 4]. However, the incidence of nucleus pulposus migration is 35%–72%, including low-migrated and high-migrated, according to the literature report [25, 26]. As for highly migrated herniations, PELD in the transforaminal approach remains challenging due to the anatomy barrier and conventional open surgery was recommended for this situation [27].

With the progress of instruments and technique, various modified techniques have been developed. Specifically, the foraminoplasty PELD has become the most commonly used technique in clinical practice, which was first introduced by Knight et al. in 1998 [28]. By using various instruments, foraminoplasty techniques could undercut the partial ventrolateral area of the superior articular process to enlarge the foramen and thus help surgeons get access to the hidden disc fragments [13]. It has been frequently reported that the foraminoplasty PELD is an effective procedure and could obtain favorable clinical outcomes for the surgical treatment of highly migrated lumbar disc herniations [13, 29]. Choi et al. [16] used reamers or endoscopic drill to widen the foramen and could approach highly migrated discs with a success rate of 94.4%.

In the current study, we compared the clinical outcomes between two-level PELD and foraminoplasty PELD for removing the highly migrated disc. These two approaches were both effective according to the operative results measured by preoperative and postoperative VAS and ODI, as well as the modified MacNab criteria.

Moreover, we also compared the postoperative complications between two-level PELD and foraminoplasty PELD. Nucleus pulposus residues were one of the most significant complications. According to the study conducted by Choi et al. [13], three of 59 patients failed to relieve symptoms due to the remnant disc material and the incidence of remnant disc material was up to 13% (7/53) in another study conducted by Kim et al. [29]. The nucleus pulposus residue is associated with the characteristic of highly migrated nucleus pulposus. Specifically, these highly migrated nucleus pulposuses are usually multifragmented and difficult to detect by preoperative radiology examination. As Kim et al. [29] reported, multifragmented nucleus pulposus was found in 19 of 53 patients. In addition, the highly migrated nucleus pulposus was easily snapped off when pulled out by surgeons. Therefore, the fragmented herniations were difficult to be completely removed just by grasping the proximal part of the herniations, which would result in missing of fragments or disconnecting from the stalk for the patients with highly migrated disc herniations.

In this study, there was only one case presented with the symptomatic residual disc in the foraminoplasty PELD group, but no nucleus pulpous residue was discovered in the two-level PELD group. During the surgical process of two-level PELD, we were able to confirm whether the migrated nucleus pulposus was completely removed through two working channels. If we found the debris nucleus pulposus shifted away, we could remove them immediately through the channels. Therefore, the two-level PELD took great advantages regarding reduction of the incidence of postoperative nucleus pulposus residue.

Another common complication was postoperative dysesthesia (POD). In our study, there was one patient in each group demonstrated with the symptoms of POD. This might be because of the special anatomical features. The foraminal dimension decreases as the spinal level decreases, and the L5/S1 level has a relatively higher iliac crest and larger facet joint and transverse process as compared to the other spinal levels, which makes the transforaminal approach for L5/S1 more difficult. The drills or reamers may irritate the exiting nerve roots during the foraminoplasty procedures or the placement of working channels. Therefore, we suggested applying the repeated fluoroscopy to confirm the correct placement of instruments, which could help to avoid the injury of nerve roots. Besides, we also suggested that all these surgical procedures should be conducted under local anesthesia, so the patients could give responses if the nerve roots were injured.

Exceptionally, the intraoperative duration of the two-level PELD technique was longer than that of the foraminoplasty technique. This is partially explained by the need of multiple punctures and placement of working channels in the two-level PELD group. In addition, another key limitation for the two-level PELD technique was its limited application in the lumbar spine, such as the high-cranially migrated herniations at the L3/4 level. It was mainly because of the special anatomic features, including short and fixed nerve roots, narrow spinal canal, and narrow lamina window for the upper lumbar disc herniations.

There were several limitations to the current study. First, the study sample size was relatively small, and thus the identified differences and statistical power should be confirmed in a larger scale cohort in consideration of different lesion levels (high-cranial versus high-caudal). Second, the current study was a retrospective study, and future prospective, randomized controlled trials with a longer follow-up period are warranted to systematically compare the clinical outcomes. However, severing as a pilot investigation, we established a system to further validate the clinical efficacy of the two-level PELD against the foraminoplasty PELD.

## 5. Conclusion

Current evidences indicate that the clinical outcomes of two-level PELD are equivalent to those of foraminoplasty PELD and these two techniques are safe and effective procedures for highly migrated lumbar disc herniations. Moreover, the two-level PELD technique has the merits in reducing the incidence of postoperative nucleus pulposus residue.

## Figures and Tables

**Table 1:** Demographic and clinical characteristics (*N* = 40).

Parameters	Two-level PELD (*N* = 14)	Foraminoplasty PELD (*N* = 26)	*P* value
Age (year)	47.3 ± 13.3	42.4 ± 9.4	0.18
Sex			
Male	5	14	0.33
Female	9	12	
Conservative time (month)	4.4 ± 1.0	4.5 ± 1.3	0.65
Intraoperative duration (minute)	80.2 ± 6.6	64.1 ± 7.3	<0.01
Follow-up time (month)	18.5 ± 3.0	17.2 ± 2.7	0.15
Location of lesions			
L3/4	5	4	0.34
L4/5	7	14	
L5/S1	2	8	

**Table 2:** Clinical outcomes and complications of two groups (*N* = 40).

Parameters	Two-level PELD (*N* = 14)	Foraminoplasty PELD (*N* = 26)	*P* value
Back VAS score			
Before operation	7.4 ± 1.9	6.9 ± 1.7	0.67
After 3 months	2.4 ± 0.9	2.1 ± 1.0	
After 12 months	1.5 ± 0.8	1.7 ± 0.8	
Final follow-up	1.1 ± 0.7	1.3 ± 0.6	
Leg VAS score			
Before operation	8.2 ± 1.5	7.7 ± 1.5	0.86
After 3 months	2.0 ± 0.7	2.4 ± 0.9	
After 12 months	1.6 ± 0.6	1.8 ± 0.8	
Final follow-up	1.1 ± 0.5	1.3 ± 0.7	
ODI score			
Before operation	60.6 ± 14.7	56.8 ± 11.2	0.87
After 3 months	29.6 ± 7.8	27.9 ± 8.4	
After 12 months	19.2 ± 4.7	21.5 ± 6.1	
Final follow-up	11.4 ± 3.2	13.2 ± 4.9	
Clinical outcome			
Excellent	5	11	0.92
Good	8	13	
Fair	1	1	
Poor	0	1	
Complications			
POD	1/14	1/26	>0.05
Disc residue	0/14	1/26	>0.05
Recurrence	0/14	1/26	>0.05

VAS: Visual Analog Scale. ODI: Oswestry Disability Index. POD: postoperative dysesthesia.

## Data Availability

The data are available via the following link: https://pan.baidu.com/s/15kZAvdR3GZe-DlpCksssow or from the corresponding author upon request.

## Supplementary Materials

Supplementary Materials Supplementary Table 1: The levels of lesions in two-level PELD group and foraminoplasty PELD group.
