# Supplementary material for: Comparison of Clinical Outcomes of Two-Level PELD and Foraminoplasty PELD for Highly Migrated Disc Herniations: A Comparative Study
Source: Biomed Res Int. 2019 Oct 13;2019:9681424. doi: 10.1155/2019/9681424 (PMC6815572; doi:10.1155/2019/9681424)
Supplement: Supplementary Materials — Supplementary Table 1: The levels of lesions in two-level PELD group and foraminoplasty PELD group. [file 9681424.f1.pdf]

Supplementary Table 1: The levels of lesions in two-level PELD group and foraminoplasty PELD group.

Variables	Two-level PELD (N=14)			Foraminoplasty PELD (N=26)		
	L3/4	L4/5	L5/S1	L3/4	L4/5	L5/S1
High-caudal	5	4	0	4	10	6
High-cranial	0	3	2	0	4	2
